# A comparative analysis of mean charts assuming Weibull and generalized exponential distributions

**DOI:** 10.1016/j.heliyon.2024.e40001

**Published:** 2024-10-31

**Authors:** Asad Raza, Sajid Ali, Ismail Shah, A.Y. Al-Rezami, Mohammed M.A. Almazah

**Affiliations:** a Department of Statistics, Quaid-i-Azam University, Islamabad 45320, Pakistan; b Department of Statistical Sciences, University of Padua, 35121, Padova, Italy; c Mathematics Department, Prince Sattam Bin Abdulaziz University, Al-Kharj 16278, Saudi Arabia; d Department of Mathematics, College of Sciences and Arts (Muhyil), King Khalid University, Muhyil 61421, Saudi Arabia

**Keywords:** Average run length, Generalized exponential distribution, Mean chart, Statistical process monitoring, Weibull distribution

## Abstract

This study discusses Shewhart control charts to monitor the mean of non-normally distributed data using extensive Monte Carlo simulations. In particular, Weibull, exponential, and generalized exponential distributions are used to conduct a systematic study. Different parameters settings are considered to evaluate the effectiveness of the Shewhart charts based on the normal approximation. The results show significant differences between the charts using generalized exponential and Weibull distributions. In particular, the generalized exponential distribution based chart achieves the desired average run length of 370 with a single threshold value across a wide range of shape parameter combinations. On the other hand, the Weibull distribution requires different threshold values depending on its shape parameter. These findings highlight the significance of the two most commonly used distributions in reliability studies and their impact on control chart performance.

## Highlights

• **Problem:** The study examines how well Shewhart control charts monitor the mean of non-normally distributed data.
• Different parameters settings are considered to evaluate the effectiveness of the Shewhart chart and normal approximation.
• The generalized exponential distribution demonstrates the stability to achieve the desired ARL of 370 for a range of parameter combinations.
• These findings highlight the significance of the two most commonly used distributions in reliability studies and their impact on control chart performance.
• **What is Already Known:** Although there have been many prior explorations related to non-normal distributions, the effectiveness of central limit theorem to design mean chart assuming generalized exponential is not discussed.
• **What This Paper Adds**: A mean control chart using a generalized exponential distribution is proposed.

## 1 Introduction

Statistical quality control methods are vital in modern productions because they help ensure that products meet both client performance criteria and regulatory standards. A quality characteristic is a metric to assess the quality of a process or product. For instance, certain processes may require specific temperature levels or durations in manufacturing. These process characteristics require continuous monitoring throughout the product's production cycle. The quality characteristics are quantifiable attributes of the product, such as its dimensions, strength, capacity, density, or weight. For example, critical product dimensions like wire thickness or the strength of a plastic component are considered quality attributes. Monitoring production involves taking samples from a process or a product and applying statistical techniques to determine if predefined standards are being met. This practice is commonly referred to as statistical process control (SPC). The purpose of SPC is to track variation in a selected quality characteristic for the product of a given process and to alert when the variation deviates from a “normal variation.” It is worth mentioning that a random variation is an integral part of any process, and it arises due to process' inherent fluctuation, or background noise by minor and unavoidable reasons. When a process only experiences random variations, it is considered to be under statistical control. Assignable causes are variations that do not fall under the category of random variations. If assignable causes of a variation exist, the process is said to be out of statistical control. A damaged gasoline pipe that raises a car's basic fuel consumption, a traumatic experience that affects a child's school performance, and sudden unemployment that affects an individual's mental health are all examples of special-cause variation.

Control charts are useful tools for minimizing process variability and for identifying when a process may be out of statistical control. Control charts are used to monitor quantities, like mean, standard deviation, range, individual observations, proportion, frequency, etc. If a process yields a high output volume, it may be more practical to take samples of size “n” at specific time intervals “t.” Then, different metrics computed from collected samples can be used to construct a control chart. A chart comprises a center line, representing the long-term average quality parameter under in-control conditions, such as the mean of the underlying process distribution or the target quality statistic. The upper and lower control limits, typically set at a distance “k” from the center line in standard deviation units, serve as thresholds to declare process in-control (IC) or out-of-control (OOC) [1]. With the technological advancements, the manufacturing processes are becoming more complex, and thus, researchers continuously proposing efficient control charts to monitor such processes.

In the existing literature, Cryer and Ryan [2] discussed the estimation of *σ* for *X* chart, highlighting the inefficiencies of the conventional approach of using moving ranges compared to sample standard deviation, particularly when dealing with correlated data. Padgett et al. [3] investigated *α*-risks associated with Shewhart control charts in quality control. Quesenberry [4] examined the impact of sample size on estimated limits for $\bar{x}$ and *X* control charts. Alwan and Roberts [5] discussed the issue of improperly positioned control limits in charts. Chou et al. [6] discussed the challenges of dealing with non-normal data in the SPC and suggested a solution to transform such data into a normal distribution using the Johnson system of distributions.

Albers and Kallenberg [7] provided simple corrections to improve the performance of control charts. They investigated the performance of control charts in the presence of mean and standard deviation estimation. They proposed one-sided and two-sided limits with corrections. Tsai et al. [8] presented a new approach for constructing control limits for $\bar{x}$ control chart when the number of subgroups is small. The proposed method uses student's t-distribution and ensures that the control limits are close to the true limits, even when only a few initial subgroups are available. Jensen et al. [9] investigated the effect of parameter estimate on an attribute control chart. The work emphasizes the importance of larger sample sizes in Phase I to attain comparable performance with known-parameters scenarios. Chakraborti [10] discussed the effect of parameter estimation for $\bar{x}$ chart. Albers and Kallenberg [11] discussed the effect of estimating parameters and the use of group statistics. They explored the behavior of control charts based on small groups and compared the performance of different group statistics. Schoonhoven and Does [12] investigated the non-normality design strategies for the $\bar{x}$ control chart. The authors analyzed numerous standard deviation estimators and examined their impact on control chart performance for non-normally data.

Psarakis et al. [13] discussed the effect of estimation on the control charts and noted that accurate parameter estimation increases the efficiency of control charts. Jones-Farmer et al. [14] provided a thorough overview of Phase I analysis for process monitoring and improvement. Faraz et al. [15] investigated the effectiveness of the $S^{2}$ control chart for estimated parameters. Chou et al. [16] proposed acceptance control charts for non-normal data for monitoring nonconforming fraction by assuming Burr distribution. Lin and Chou [17] discussed the performance of the variable sample size and control limits $\overset{¯}{X}$ chart using Burr distribution. Khakifirooz et al. [18] pointed out the problems using nonlinear transformations to obtain the approximate normality. Tsai [19] developed two Shewhart-type charts and process capability ratios using the skew normal distribution to monitor the process average and evaluate the process capability. Lin and Chou [20] discussed the performance of variable parameters $\overset{¯}{X}$ for non-normal data and further compared to the adaptive chart. It is shown that the variable parameters chart outperforms the competitors. Chen and Yeh [21] discussed the economic design of $\overset{¯}{X}$ chart assuming Burr distribution under Weibull failure mechanism. Chen and Cheng [22] discussed economic–statistical design of $\overset{¯}{X}$-control charts using Johnson distribution.

The correction factors to obtain the unconditional average run length (ARL) for Shewhart control charts are discussed in Goedhart et al. [23]. Goedhart et al. [24] discussed a method for modifying the control limits of Shewhart control charts to monitor process variability. Goedhart et al. [25] presented two methods for determining adjusted control limits for the Shewhart *X* and $\bar{X}$ control charts to ensure a specified IC performance. The first method is based on the bootstrap approach, while the second method is an analytical approach. Huberts et al. [26] applied the central limit theorem to evaluate the performance of a Shewhart control chart for large non-normally distributed datasets for various distributions and sample sizes. Durtka [27] studied the ARL properties for skewed data. [28] proposed bootstrap control chart for monitoring the percentile of Weibull distribution. We refer the interested readers to Erto and Pallotta [29], Faraz et al. [30], Vasconcelos et al. [31], Ho et al. [32], Derya and Canan [33], Huang et al. [34], Ali and Shah [35], Ali [36], Ali and Pievatolo [37], Huberts et al. [26], and references cited therein for control charts based on non-normal distributions.

The volume and frequency of data generated by different businesses have grown dramatically during the last few decades. At the same time, computing capacity has also expanded dramatically. However, when it comes to the distribution of data, the conventional assumption of normality is challenged. Thus, a comprehensive investigation is required to evaluate the usefulness and adaptability of Shewhart charts using non-normal models. Thus, this study investigates the Shewhart control chart [38] under the assumption of asymmetrical, non-normally distributed sample data to monitor the mean. To assess the performance of the charts, the ARL is used. To evaluate the performance of the charts using Weibull and generalized exponential distributions, a detailed Monte Carlo study is conducted. We use a wide range of shifts and sample sizes to monitor the two most important non-normal distributions that regularly arise in reliability theory.

The rest of the article is organized as follows. Section 2 presents Shewhart charts for monitoring the mean of Weibull and generalized exponential distributions. The performance assessment of the charts using the ARL to verify the applicability of central limit theorem is discussed in Section 3. Finally, Section 4 concludes the study.

## 2 Shewhart charts for Weibull and generalized exponential distribution

The R and $\bar{x}$ charts are the most frequently used Shewhart charts. The $\bar{x}$ chart, also known as the control chart for the mean, tracks the mean or average value of the selected quality characteristic. The sample mean, $\bar{x_{i}}$, is plotted on the $\overset{¯}{x}$ control chart at time *j*, and computed as

(1) $$\bar{x_{j}}=\frac{x_{j1}+x_{j2}+\ldots +x_{jn}}{n},$$

where $x_{ji}$, j=1,2,..., i=1,2..., n, in Eq. (1) represents the $i^{\text{th}}$ observation in the sample of size *n* at time *j*. If the obtained sample mean falls between the control limits, the process is assumed to be IC. If a sample is plotted beyond the control bounds, it is considered to be out-of-control, and the source of the special cause must be investigated.

The midline of the $\bar{x}$ chart indicates the IC mean of the process, denoted as $\mu_{0}$. The upper control limit (UCL) and the lower control limit (LCL) are

(2) $$UCL=\mu_{0}+k\cdot \frac{\sigma_{0}}{\sqrt{n}}$$

(3) $$LCL=\mu_{0}-k\cdot \frac{\sigma_{0}}{\sqrt{n}}$$

where $\sigma_{0}$ represents the in-control standard deviation, and the parameter ‘k,’ controls the width of control limits, is often selected to indicate substantial process changes [1]. Slight changes in the value of *h* or an increase in the sample size will affect the ARL and the likelihood of detection.

It is a well-known fact that the distribution of the run length assuming three sigma control limits is a geometric distribution [1]. Then, assuming normality of quality characteristics, the probability of OC is 0.002699796. Therefore, the ARL is just the expected value of a geometric distribution, which is 370.4 in this case.

### 2.1 Weibull distribution

The Weibull distribution is a continuous lifetime probability distribution and used to model the occurrence of failures for assessing the reliability of products. This distribution is widely used in many fields, including economics, hydrology, biology, and engineering sciences [39]. The probability density function of a Weibull distribution is

(4) $$f(x;\gamma ,\lambda )=\left\{ \begin{array}{cc} \gamma \lambda {\left(x\lambda \right)}^{\gamma -1}e^{-{\left(x\lambda \right)}^{\gamma }}, & x>0\\ 0, & x<0 \end{array}\right.$$

If the shape parameter *γ* is less than 1, it signifies that the failure rate decreases over time. This leads to “infant mortality,” where items tend to fail lately, leading to a decreasing failure rate. However, if the shape parameter *γ* is equal to 1, it indicates a constant failure rate over time like the exponential distribution. Similarly, a value of the shape parameter *γ* greater than 1 implies that the failure rate increases with time. This phenomenon can be attributed to an “aging” process or the presence of components that become more prone to failure as time progresses.

The mean of the Weibull distribution with *γ* and *λ* is

(5) $$\mu =\frac{1}{\lambda }\cdot \Gamma \left(1+\frac{1}{\gamma }\right)$$

while the variance is

(6) $$\sigma^{2}=\frac{1}{\lambda^{2}}\left[\Gamma \left(1+\frac{2}{\gamma }\right)-{\left(\Gamma \left(1+\frac{1}{\gamma }\right)\right)}^{2}\right]$$

### 2.2 Generalized exponential distribution

Gupta and Kundu [40] discussed two-parameter generalized exponential distribution for lifetime data. The functional form of the distribution is very similar to the two-parameter gamma or Weibull distribution but more flexible for analyzing skewed data. Specifically, if the shape parameter *γ* is set to one, it reduces to the one-parameter exponential distribution, making it an extension or generalization of the one-parameter exponential distribution. Besides functionality and flexibility, the distribution also has practical interpretations. For instance, in a parallel system composed of *n* components, where the system operates as long as at least one component functions, the system's lifetime distribution can be expressed as

(7) $$F(x;n,\lambda )={\left(1-e^{-x\lambda }\right)}^{n},\;x>0.$$

The density function of the generalized exponential distribution derived from Eq. (7) is given by

(8) $$f(x;\gamma ,\lambda )=\gamma \lambda {\left(1-e^{-x\lambda }\right)}^{\gamma -1}e^{-x\lambda },\;x>0$$

where both *γ* and *λ* are greater than zero. The density function exhibits various shapes. When *γ* is less than or equal to one, the function decreases, and for *γ* greater than one, it forms a unimodal, skewed, right-tailed distribution similar to the Weibull or gamma density function. The effect of the shape parameter is very similar as discussed in the case of Weibull distribution. It is worth noting that even for very large values of the shape parameter, the distribution remains asymmetric. The expected value of a generalized exponential distribution is

(9) $$E(X)=\frac{1}{\lambda }[\psi (\gamma +1)-\psi (1)]$$

while the variance is

(10) $$V(X)=\frac{1}{\lambda^{2}}[\psi^{\prime }(1)-\psi^{\prime }(\gamma +1)]$$

where *ψ*(.) represents the digamma function, and $\psi^{\prime }$(.) is the trigamma function, which are the first and second derivatives of the gamma function, respectively. It is important to mention that as *λ* decreases, the mean of a generalized exponential distribution approaches to infinity. Similarly, for a fixed *β*, the variance tends to $\frac{\pi^{2}}{6\lambda }$.

## 3 Performance assessment

This section assesses the performance of Shewhart charts assuming Weibull and generalized exponential distributions to monitor non-normally distributed data. To this end, we use Monte Carlo study to achieve the pre-fixed ARL by considering various sample sizes “n”, shifts, and the number of simulation runs “m”. The shifts in the process rate parameter are defined as

(11) $$\delta =\frac{\sqrt{n}\left|\lambda -\lambda_{0}\right|}{\sigma }$$

where *n* is the sample size, *σ* is the process standard deviation, *λ* is the shifted process rate parameter, and $\lambda_{0}$ is the in-control process rate parameter. The absolute values are used because the rate parameter is always positive in Weibull and generalized exponential distributions, respectively. A shift of δ=1 indicates that the process mean has shifted one standard error away from the target mean.

The R programming language is used to calculate the ARL for different scenarios. The steps to calculate the ARL are given below.

(1) Using the in-control parameters, calculate the mean and standard deviation.
(2) Then, using the given sample size and an arbitrary value of *k*, establish control limits.
(3) Generated out-of-control random samples by introducing shifts and calculated the mean to plot against the established control limits.
(4) If the mean falls within the control limit, the process is declared in-control.
(5) If the computed mean does not fall within the control limits, a signal of out-of-control is recorded and stored in the run length vector.
(6) Repeating *m* times steps 3-5. Then, the average of the run length vector is calculated to obtain the ARL.

It is worth mentioning that a simulation run is discarded if no out-of-control signal is recorded. If the desired ARL is not achieved with a very large sample size, the value of *k* is modified.

For the Weibull distribution, the analysis is conducted assuming three different *γ* combinations (0.8, 1, 1.5) with a constant rate (*λ*) parameter of one. It is worth mentioning that the shape parameters are selected to represent decreasing, constant, and increasing failure rates [37], [36] in both distributions. The objective is to achieve the ARL target of 370, acting as a reference for assessing control chart performance. To evaluate the effect of shifts, shifts ranging from 0.0 to 3.0 are introduced and to achieve the desired ARL value of 370, adjustments are made to the width parameter *k*. More specifically, for the generalized exponential distributions with *γ* and *λ* parameter combinations (0.8, 1), (1, 1), and (1.5, 1), the control chart distance threshold (k) is set at 3, approximating an IC ARL of 370 as the number of observations (n) approaches 1000. However, for the Weibull distribution for the aforementioned combinations, different *k* values are employed to achieve the desired ARL. In particular, for *γ* = 0.5 and *λ* = 1, k is set at 3.6, while for *γ* = 1 and *λ* = 1, k is set at 3, and for *γ* = 1.5 and *λ* = 1, k is set at 2.33.

### 3.1 Weibull with *γ* = 0.8, *λ* = 1, and *k* = 3.6

To explore different combinations of the number of observations (*n*) and number of simulations (*m*) under various *δ* scenarios, Table 1 lists the ARL values calculated for the Weibull distribution with *γ* = 0.8, *λ* = 1, and *k* = 3.6. For IC scenarios, the ARL should closely align with a target value, which is 370 in this study. In particular, the table examines different number of simulations (30, 50, 100, 200, 1000, 5000, 10000) and *δ* values for a given number of observations *n* = 5. Assuming *δ* = 0, the ARL values for simulations 30, 50, 100, and 200 are 88.67, 84.36, 84.44, and 78.73, respectively. For a shift of size 0.2, the ARL values decrease to 45.97, 40.52, 45.69, and 48.29, signifying increased sensitivity of the control chart for detecting process mean shifts. With *δ* = 1.0, the ARL values decrease further (10.13, 11.34, 10.44, 9.40). However, for a *δ* of 2.0, the ARL values remain relatively consistent (3.60, 3.92, 3.83, 4.19), indicating sustained sensitivity. A *δ* of 3.0 results in ARL reductions (2.93, 2.28, 2.11, 2.59), demonstrating the evolving ability to detect *δ*.

**Table 1:** ARL values for different combinations of *n*, *m*, and *δ* for Weibull distribution with *γ* = 0.8 and *λ* = 1.

		Weibull distribution (γ=0.8,λ=1,k=3.6)						
	*m*	30	50	100	200	1000	5000	10000
*n*	*δ*	ARL	ARL	ARL	ARL	ARL	ARL	ARL
5	0.0	88.67	84.36	84.44	78.73	81.09	79.03	79.67
	0.2	45.97	40.52	45.69	48.29	43.35	44.74	45.26
	0.4	25.33	23.08	22.59	27.75	27.06	27.14	27.86
	0.6	19.30	17.14	17.54	19.13	19.61	18.72	18.84
	0.8	18.13	15.04	14.42	11.69	14.00	13.36	13.50
	1.0	10.13	11.34	10.44	9.40	10.26	10.24	10.00
	1.2	7.73	9.32	5.89	7.94	8.10	8.22	7.94
	1.4	6.13	5.94	7.71	7.51	6.77	6.44	6.57
	1.6	4.40	5.74	4.95	5.22	5.39	5.50	5.56
	1.8	5.33	3.94	4.25	4.25	4.99	4.66	4.73
	2.0	3.60	3.92	3.83	4.19	4.07	4.19	4.19
	2.2	4.43	3.44	3.50	3.86	3.59	3.64	3.63
	2.4	3.57	2.92	3.02	3.16	3.20	3.26	3.31
	2.6	2.90	3.12	2.81	3.10	2.90	3.03	2.98
	2.8	3.67	2.86	2.55	2.68	2.76	2.74	2.73
	3.0	2.93	2.28	2.11	2.59	2.61	2.56	2.53
30	0.0	268.30	169.90	209.39	175.38	181.17	184.22	179.93
	0.2	108.77	85.66	107.89	101.88	99.65	101.51	102.90
	0.4	55.70	72.30	58.49	57.06	61.38	59.47	60.89
	0.6	37.07	42.32	45.29	39.47	37.53	38.26	38.46
	0.8	28.63	23.02	21.17	26.30	24.59	25.13	25.40
	1.0	19.60	17.68	18.36	17.97	18.67	17.69	17.61
	1.2	13.07	9.50	11.97	14.11	12.88	12.79	12.56
	1.4	9.63	12.00	9.57	8.79	9.83	9.62	9.63
	1.6	8.97	7.24	9.12	7.14	7.44	7.57	7.43
	1.8	6.80	5.70	6.21	5.68	5.79	5.79	5.99
	2.0	4.83	4.30	5.22	4.84	4.90	4.85	4.85
	2.2	4.33	4.44	4.50	4.12	4.06	4.03	4.02
	2.4	3.97	3.60	3.38	3.42	3.38	3.42	3.42
	2.6	2.93	2.88	2.95	3.02	3.00	2.99	2.98
	2.8	2.80	2.56	2.04	2.46	2.80	2.60	2.61
	3.0	2.57	2.72	2.11	2.14	2.43	2.35	2.33
50	0.0	202.37	232.56	213.67	246.02	222.58	226.64	223.62
	0.2	148.50	117.58	132.77	146.56	125.74	126.35	126.46
	0.4	101.03	87.32	71.01	73.71	73.21	72.43	74.31
	0.6	58.20	46.24	48.94	47.77	46.08	46.09	46.60
	0.8	27.57	31.00	30.66	25.80	29.47	29.90	30.60
	1.0	24.23	22.64	20.25	20.70	19.96	20.32	20.82
	1.2	15.77	14.68	14.81	14.66	15.37	14.66	14.56
	1.4	11.83	12.64	9.57	10.36	10.56	10.86	10.80
	1.6	7.77	9.54	8.11	8.65	7.93	8.27	8.09
	1.8	5.23	6.62	5.75	6.88	6.27	6.44	6.38
	2.0	6.07	4.78	5.12	4.57	5.40	5.10	5.14
	2.2	4.30	4.52	3.84	4.39	4.37	4.20	4.20
	2.4	3.73	4.46	3.63	3.82	3.46	3.51	3.53
	2.6	3.33	2.48	3.19	2.89	3.11	2.96	3.03
	2.8	2.43	2.96	2.77	2.63	2.62	2.64	2.63
	3.0	2.60	2.26	2.43	2.46	2.29	2.34	2.31
100	0.00	247.37	271.74	262.52	264.29	281.68	282.66	280.26
	0.2	194.77	138.44	165.20	173.34	161.21	165.63	160.08
	0.4	99.17	121.22	90.30	94.75	100.23	94.32	94.28
	0.6	72.30	60.58	73.24	55.08	63.89	58.90	58.49
	0.8	34.20	35.92	36.72	44.02	38.07	37.51	36.90
	1.0	32.70	17.70	26.88	24.91	25.09	25.16	25.05
	1.2	13.17	18.62	18.73	17.96	17.40	17.55	17.21
	1.4	9.87	13.70	12.54	13.19	12.40	12.74	12.49
	1.6	9.70	10.22	8.93	8.89	8.93	9.34	9.19
	1.8	5.43	6.96	7.02	7.05	7.22	7.02	7.14
	2.0	6.90	5.92	5.79	5.77	5.46	5.53	5.56
	2.2	5.03	5.02	3.84	4.39	4.49	4.49	4.42
	2.4	4.00	3.52	4.16	3.72	3.82	3.69	3.69
	2.6	2.67	3.48	3.55	3.03	3.15	3.11	3.10
	2.8	2.63	2.86	2.89	2.68	2.62	2.63	2.63
	3.0	2.03	2.20	2.32	2.13	2.30	2.33	2.31
250	0.0	287.93	299.74	348.01	355.79	351.34	329.32	336.47
	0.2	215.23	215.78	189.58	183.11	198.44	212.68	205.78
	0.4	129.37	117.98	122.08	144.18	127.15	124.09	125.38
	0.6	87.83	82.76	77.70	80.57	77.39	76.21	75.56
	0.8	40.30	58.42	47.24	44.51	46.76	47.46	47.54
	1.0	28.53	40.00	26.49	32.55	29.92	30.83	31.50
	1.2	19.93	19.04	19.02	20.95	20.16	21.19	20.85
	1.4	18.10	14.24	14.13	13.83	14.97	14.72	15.05
	1.6	10.47	11.48	13.08	10.75	10.68	10.65	10.58
	1.8	8.03	7.60	7.20	8.43	8.15	7.73	7.89
	2.0	5.30	4.76	6.50	6.34	5.78	6.14	5.99
	2.2	5.43	5.16	4.13	4.64	4.64	4.69	4.75
	2.4	4.07	4.52	3.72	4.41	3.74	3.84	3.87
	2.6	2.83	2.94	3.37	3.26	3.07	3.10	3.17
	2.8	2.40	2.24	3.10	2.89	2.68	2.68	2.72
	3.0	2.33	2.34	2.15	2.44	2.32	2.26	2.31
1000	0.00	371.53	366.42	369.37	367.26	363.96	361.54	371.71
	0.2	235.07	255.20	259.48	226.98	255.13	257.07	264.42
	0.4	148.50	152.84	185.83	165.32	166.13	162.99	162.65
	0.6	108.00	82.24	95.65	93.18	93.76	96.06	98.48
	0.8	61.20	46.28	74.64	60.50	59.94	60.79	60.56
	1.0	37.60	42.26	36.42	36.87	38.03	38.95	38.52
	1.2	25.23	26.56	26.99	23.86	24.57	25.65	25.80
	1.4	18.40	19.36	17.36	17.01	17.52	17.66	17.54
	1.6	10.60	14.58	13.36	10.89	12.51	12.28	12.23
	1.8	8.03	10.26	8.81	10.14	9.23	8.70	9.02
	2.00	5.80	8.50	7.08	6.51	6.67	6.51	6.80
	2.2	5.53	5.60	5.02	5.64	4.89	5.13	5.14
	2.4	4.10	3.98	4.21	3.89	4.16	4.02	4.01
	2.6	3.43	3.24	3.09	3.51	3.43	3.32	3.28
	2.8	2.57	2.78	2.99	2.87	2.79	2.74	2.72
	3.0	2.60	2.74	2.37	2.42	2.29	2.28	2.30

Considering *n* = 30 and *δ* = 0, the ARL values for different simulations are 268.30, 169.90, 209.39, and 175.38. As *δ* increased to 0.2, the ARL values decreased to 108.77, 85.66, 107.89, and 101.88, indicating increased sensitivity of the control chart to detect shifts. A δ=1.0 results in further ARL reductions (19.60, 17.68, 18.36, 17.97). For the in-control case, the ARL values for m=30, 50, 100, and 200 with *n* = 50, are 202.37, 232.56, 213.67, and 246.02, respectively, whereas, for δ=0.2, the ARL values are to 148.50, 117.58, 132.77, and 146.56, respectively. With *δ*=1.0, the ARL values further decrease (24.23, 22.64, 20.25, 20.70). A *δ* of 3.0 leads to further ARL decreases (2.60, 2.26, 2.43, 2.46), highlighting the evolving capability to detect *δ*.

The ARL values for *n* = 100 and *m*=30, 50, 100, and 200 assuming *δ* = 0, are 247.37, 271.74, 262.52, and 264.29, respectively. As the *δ* increases to 0.2, the ARL values are 194.77, 138.44, 165.20, and 173.34. With *δ*=1.0 and δ=2.0, the ARL values further decrease (32.70, 17.70, 26.88, 24.91) and (6.90, 5.92, 5.79, 5.77). A *δ* of 3.0 leads to (2.03, 2.20, 2.32, 2.13). Considering *n* = 250 and *δ* = 0, the ARL values for *m*=30, 50, 100, and 200 are 287.93, 299.74, 348.01, and 355.79, respectively. As the *δ* increased to 0.2, the ARL values decreased to 215.23, 215.78, 189.58, and 183.11. Increasing *n* to 1000 results in the ARL values surpassing those observed with n=250 because as the sample size increases to 1000, the behavior of the control chart tends to follow the asymptotic normality. This is an application of the central limit theorem, which states that as the number of observations become sufficiently large, the distribution of sample means approaches to a normal distribution. For *δ* = 0, the ARL values for m=30, 50, 100, and 200 are 371.53, 366.42, 369.37, and 367.26, respectively. As *δ* increases to 0.2, the ARL values decrease to 235.07, 255.20, 259.48, and 226.98, indicating an increased sensitivity in the control chart's ability to detect *δ*. With *δ*=1.0, the ARL values further decrease (37.60, 42.26, 36.42, 36.87). Notably, with *n* = 1000, the ARL values approach the target of 370, suggesting that larger number of observations contributes to a more balanced control chart in terms of sensitivity and stability. As *δ* increases, the ARL values consistently decrease, suggesting that the control chart becomes more sensitive to out-of-control situations.

### 3.2 Weibull with *γ* = 1, *λ* = 1, and *k* = 3

Table 2 tabulates the ARL results corresponding to the Weibull distribution with *γ* = 1, *λ* = 1, *k* = 3, and this case is equivalent to the exponential distribution. With *δ* = 0 and n=5, the control chart has the ARL values of 132.63, 114.12, 103.83, and 110.97 for *m*=30, 50, 100, and 200, respectively. As the shift magnitude increases, the ARL values significantly decrease. For instance, with a δ=1.00, the ARL reduces to 10.00, 11.24, 11.85, and 9.47. Further increase in *δ* magnitude (δ>1.00) will further reduce the ARL values. As noticed previously, when *δ* is at 0, indicating the IC state, the ARL values stand at 303.60, 267.32, 265.16, and 249.05 for *m* equals to 30, 50, 100, and 200, respectively. These values imply that, on average, a considerable number of observations are required to achieve the IC ARL=370. By increasing *δ* (δ>0), the ARL values declined. For instance, *δ* = 1.00 results in the ARL values of 16.23, 21.00, 17.69, and 18.46 for the corresponding *m* values.

**Table 2:** ARL values for different combinations of *n*, *m*, and *δ* for Weibull distribution with *γ* = 1 and *λ* = 1.

		Weibull distribution (γ=1,λ=1,k=3)						
	*m*	30	50	100	200	1000	5000	10000
*n*	*δ*	ARL	ARL	ARL	ARL	ARL	ARL	ARL
5	0.0	132.63	114.12	103.83	110.97	102.96	108.09	108.25
	0.2	60.57	60.72	49.85	56.31	56.80	55.33	55.92
	0.4	40.63	33.70	39.93	33.94	33.95	32.69	33.10
	0.6	13.83	19.72	19.86	19.48	20.95	20.96	21.00
	0.8	15.23	15.16	14.98	13.74	14.24	14.47	14.45
	1.0	10.00	11.24	11.85	9.47	10.58	10.74	10.55
	1.2	9.13	7.92	6.83	6.71	8.24	8.19	8.05
	1.4	7.97	6.44	6.87	6.63	6.45	6.48	6.40
	1.6	5.03	5.46	5.26	5.43	5.28	5.20	5.32
	1.8	3.90	4.98	4.92	3.95	4.32	4.53	4.42
	2.0	4.57	3.82	4.13	4.32	3.87	3.90	3.79
	2.2	3.20	3.14	3.18	3.31	3.33	3.32	3.34
	2.4	2.67	2.46	2.82	3.08	3.06	3.00	2.95
	2.6	3.57	2.50	2.69	2.63	2.73	2.70	2.68
	2.8	2.10	2.06	2.43	2.40	2.42	2.50	2.50
	3.0	2.37	2.42	2.18	2.32	2.25	2.24	2.26
30	0.0	303.60	267.32	265.16	249.05	240.63	230.01	234.87
	0.2	109.47	129.30	126.36	145.44	116.45	123.66	125.50
	0.4	73.80	84.72	71.21	61.03	74.12	70.81	71.16
	0.6	30.43	41.70	41.10	46.05	39.24	42.39	42.63
	0.8	20.80	29.10	30.26	27.61	27.23	27.89	27.05
	1.0	16.23	21.00	17.69	18.46	17.87	18.36	18.26
	1.2	10.50	14.10	12.47	15.04	12.48	12.65	13.07
	1.4	6.03	9.06	8.75	9.38	9.64	9.26	9.49
	1.6	7.47	7.24	7.97	7.18	7.02	7.16	7.19
	1.8	6.13	5.38	6.22	5.45	5.52	5.54	5.67
	2.0	3.57	4.28	4.37	4.80	4.67	4.43	4.47
	2.2	4.23	3.92	3.43	3.88	3.71	3.69	3.73
	2.4	2.73	3.22	3.28	2.95	3.11	3.14	3.17
	2.6	3.47	2.76	2.85	2.78	2.73	2.69	2.66
	2.8	2.00	2.22	2.42	2.36	2.31	2.40	2.36
	3.0	1.77	2.14	2.03	2.15	2.05	2.11	2.08
50	0.0	266.20	163.36	262.63	284.09	280.12	279.15	268.58
	0.2	163.93	157.96	133.88	156.48	152.89	148.19	151.04
	0.4	81.77	82.28	84.05	84.84	85.81	86.62	85.55
	0.6	49.50	61.48	49.86	48.80	53.49	50.84	50.68
	0.8	24.30	25.80	30.21	33.30	31.29	32.63	31.77
	1.0	17.47	21.46	21.27	21.47	21.47	20.49	21.02
	1.2	19.03	15.72	12.22	14.24	15.50	14.66	14.56
	1.4	10.67	13.64	8.27	10.96	10.04	10.40	10.45
	1.6	4.80	8.14	6.98	7.36	7.61	7.87	7.65
	1.8	5.87	5.58	5.66	5.74	5.72	5.91	5.96
	2.0	3.10	4.52	4.64	4.84	4.91	4.76	4.65
	2.2	4.17	4.02	3.45	3.76	3.60	3.84	3.83
	2.4	3.03	3.12	2.93	3.12	3.10	3.18	3.22
	2.6	2.27	2.58	2.61	2.53	2.76	2.74	2.72
	2.8	2.43	2.50	2.98	2.19	2.30	2.34	2.35
	3.0	2.17	1.98	2.11	2.10	2.05	2.09	2.08
100	0.0	332.23	362.00	298.90	294.69	309.96	308.55	312.92
	0.2	209.33	217.86	186.38	176.99	188.18	185.25	181.64
	0.4	98.30	103.94	104.16	120.39	100.08	104.55	105.82
	0.6	73.43	61.02	58.49	61.12	62.55	62.56	62.41
	0.8	29.60	44.64	40.34	38.96	39.02	38.23	39.35
	1.0	19.80	20.88	23.63	25.38	24.69	25.41	25.01
	1.2	13.63	17.64	19.03	18.00	16.92	16.77	17.02
	1.4	10.07	10.52	11.45	11.59	11.67	12.02	12.04
	1.6	8.33	8.68	8.77	8.50	8.55	8.49	8.51
	1.8	6.20	6.78	6.46	6.83	6.53	6.38	6.50
	2.0	4.40	5.26	5.81	5.22	5.04	4.97	5.05
	2.2	4.50	4.08	3.91	3.91	3.92	4.03	3.96
	2.4	4.20	3.34	3.48	3.38	3.46	3.25	3.26
	2.6	2.23	3.10	2.66	2.57	2.74	2.76	2.71
	2.8	2.27	2.12	2.54	2.15	2.44	2.38	2.36
	3.0	2.57	2.40	2.10	2.03	1.96	2.03	2.07
250	0.0	330.40	334.36	268.43	320.10	354.04	338.76	350.51
	0.2	273.93	194.96	246.35	199.92	223.67	223.42	223.57
	0.4	150.57	136.34	126.06	136.69	132.24	130.97	131.05
	0.6	83.17	88.44	68.24	74.96	79.73	78.28	78.00
	0.8	43.77	35.32	48.65	44.21	48.58	46.74	46.25
	1.0	38.90	22.86	30.80	30.85	31.09	30.29	30.13
	1.2	22.70	19.68	17.33	20.78	19.95	19.59	19.50
	1.4	11.00	13.40	13.16	13.65	13.07	13.56	13.65
	1.6	10.57	8.32	9.83	10.35	9.54	9.80	9.66
	1.8	8.60	9.10	8.03	9.11	7.31	6.96	6.97
	2.0	5.37	5.16	6.32	6.01	5.30	5.21	5.32
	2.2	4.17	4.54	3.80	4.22	4.38	4.31	4.15
	2.4	2.90	3.54	3.36	3.16	3.37	3.35	3.36
	2.6	3.43	2.76	2.86	2.63	2.73	2.74	2.80
	2.8	2.03	2.00	2.20	2.17	2.39	2.38	2.37
	3.0	2.40	2.32	1.97	2.01	2.09	2.03	2.01
1000	0.0	342.77	361.94	370.43	362.74	363.97	368.97	369.75
	0.2	293.03	249.20	258.90	251.59	252.15	257.70	258.93
	0.4	88.43	161.26	157.19	164.66	155.44	155.66	158.18
	0.6	76.63	92.26	95.63	88.50	90.56	94.37	96.62
	0.8	49.17	63.80	53.51	51.72	58.21	56.99	57.49
	1.0	39.57	35.00	36.14	36.76	34.45	35.24	35.53
	1.2	22.30	32.00	21.58	23.22	23.94	22.95	23.12
	1.4	16.87	14.06	15.42	16.04	16.14	15.52	15.44
	1.6	8.43	8.48	12.27	9.64	10.40	10.88	10.75
	1.8	7.53	7.22	7.36	7.42	7.74	7.68	7.61
	2.0	8.40	4.76	7.03	5.17	5.65	5.82	5.64
	2.2	4.37	4.24	4.45	4.68	4.37	4.43	4.39
	2.4	4.67	4.20	3.06	3.36	3.53	3.45	3.49
	2.6	2.60	2.82	2.79	3.14	2.71	2.82	2.84
	2.8	2.50	2.46	2.33	2.30	2.43	2.37	2.36
	3.0	1.87	1.74	2.02	2.01	2.06	1.99	2.01

For n=50, the IC (*δ* = 0) ARL values are 266.20, 263.36, 262.63, and 284.09 for *m*=30, 50, 100, and 200, respectively, indicating a need for a substantial number of samples to achieve the desired ARL. For instance, with *δ* = 1.00, the ARL values decrease to 17.47, 21.46, 21.27, and 21.47 across the corresponding *m* values. Further increments in shift magnitude (δ>1.00) result in further decreased ARL values. For *n* = 100, the IC case (*δ* = 0) has the ARL values of 332.23, 362.00, 298.90, and 294.69 for *m* = 30, 50, 100, and 200, respectively, suggesting that a large sample size should be used to achieve the desired IC ARL. Assuming *n* = 250 and *δ* = 0, the ARL values are 330.40, 334.36, 268.43, and 320.10 for *m* = 30, 50, 100, and 200, respectively. The chart with *δ* = 0 and *n* = 1000 has the ARL values of 342.77, 361.94, 370.43, and 362.74 for *m* = 30, 50, 100, and 200, respectively, indicating that n=1000 is sufficient to achieve the desired ARL. The diminishing ARL values with increasing shift magnitudes (δ>0) underscore the increased sensitivity of the control chart in detecting shifts in the process. With *δ* = 1.00, the ARL values decrease to 39.57, 35.00, 36.14, and 36.76 across the corresponding *m* values, indicating a swifter response to OOC conditions. Similarly, shifts δ>1.00 result in further decreased ARL values, highlighting the chart's ability to promptly identify deviations from the in-control state.

### 3.3 Weibull with *γ* = 1.5, *λ* = 1, *k* = 2.33

Table 3 lists the ARL results for the Weibull distribution with *γ* = 1.5 and *λ* = 1. Using *k* = 2.33, different numbers of simulations (30, 50, 100, 200, 1000, 5000, 10000) are examined to observe their influence on the ARL values. In the absence of a shift (*δ* = 0) with *n* = 5, the ARL values for sample sizes 30, 50, 100, and 200 are 193.90, 220.02, 190.77, and 202.37, respectively. The presence of a shift (δ>0), such as a *δ* = 1.00, results in reduced ARL values (9.70, 11.30, 10.19, 9.99). On exploring further, a *δ* = 2.00 leads to further reduced ARL (3.43, 2.70, 3.34, 3.39). The primary objective is to achieve a target ARL of 370, potentially requiring a large sample sizes to maintain an optimal balance between sensitivity and stability.

**Table 3:** ARL values for different combinations of *n*, *m*, and *δ* for Weibull distribution with *γ* = 1.5 and *λ* = 1.

		Weibull distribution (γ=1.5,λ=1,k=2.33)						
	*m*	30	50	100	200	1000	5000	10000
*n*	*δ*	ARL	ARL	ARL	ARL	ARL	ARL	ARL
5	0.0	193.90	220.02	190.77	202.37	199.73	189.40	197.50
	0.2	96.13	87.30	90.19	69.68	84.83	86.73	85.44
	0.4	31.93	45.56	40.24	41.84	41.79	42.15	43.44
	0.6	21.87	24.58	23.36	22.03	24.52	24.61	24.66
	0.8	14.13	14.54	13.65	14.43	14.91	15.42	15.43
	1.0	9.70	11.30	10.19	9.99	10.96	10.32	10.54
	1.2	9.87	6.38	7.26	6.75	7.74	7.60	7.57
	1.4	5.80	5.20	5.84	6.06	5.70	5.68	5.68
	1.6	5.03	4.10	4.26	4.13	4.65	4.54	4.53
	1.8	3.30	3.24	3.72	3.64	3.68	3.68	3.69
	2.0	3.43	2.70	3.34	3.39	3.06	3.08	3.09
	2.2	2.70	2.68	2.19	2.88	2.59	2.72	2.67
	2.4	2.23	2.78	2.26	2.41	2.38	2.37	2.40
	2.6	1.70	2.00	1.90	2.18	2.13	2.15	2.13
	2.8	1.60	1.70	1.66	1.89	1.92	1.92	1.94
	3.0	1.83	1.52	1.90	1.66	1.81	1.81	1.79
30	0.0	214.13	267.22	241.56	320.85	309.70	316.73	315.80
	0.2	160.57	139.98	168.76	146.06	161.38	158.45	162.57
	0.4	64.87	97.70	102.84	86.59	83.45	84.07	82.69
	0.6	34.53	38.76	40.56	46.07	46.35	46.00	46.21
	0.8	20.70	25.72	25.08	26.09	27.36	26.41	26.51
	1.0	16.73	17.58	14.53	16.18	17.37	16.39	16.95
	1.2	11.47	9.56	11.49	10.17	11.48	11.31	11.01
	1.4	7.33	10.04	7.88	8.62	8.26	7.96	7.81
	1.6	5.77	5.78	4.96	6.20	5.88	5.82	5.78
	1.8	6.63	3.44	4.48	4.09	4.54	4.37	4.36
	2.0	3.70	2.78	3.44	3.81	3.38	3.44	3.40
	2.2	2.37	2.64	2.65	2.80	2.75	2.86	2.78
	2.4	2.77	2.42	2.53	2.42	2.46	2.38	2.36
	2.6	2.03	2.12	1.94	2.00	2.04	2.08	2.06
	2.8	1.73	1.76	1.75	1.69	1.79	1.80	1.81
	3.0	1.77	1.40	1.59	1.62	1.67	1.61	1.61
50	0.0	252.33	367.98	314.41	312.59	329.13	325.07	329.89
	0.2	148.43	173.36	172.95	164.02	172.30	183.72	179.82
	0.4	87.50	87.14	95.39	98.37	93.32	98.08	94.99
	0.6	51.67	51.58	56.38	54.41	54.22	53.15	51.73
	0.8	30.63	31.76	29.26	29.16	30.50	30.11	30.52
	1.0	17.17	20.38	20.39	19.89	18.15	18.60	18.88
	1.2	12.03	10.66	11.90	12.55	12.37	12.39	12.26
	1.4	6.83	7.50	7.64	8.56	8.68	8.36	8.49
	1.6	7.07	5.40	6.24	6.18	5.99	6.00	5.94
	1.8	3.90	4.46	4.34	4.34	4.33	4.56	4.52
	2.0	4.27	3.26	4.00	3.41	3.69	3.53	3.58
	2.2	3.30	2.66	2.66	2.70	2.92	2.84	2.85
	2.4	1.93	2.34	2.21	2.18	2.36	2.35	2.36
	2.6	1.77	2.22	1.90	2.15	1.97	2.06	2.02
	2.8	1.73	1.86	1.69	1.83	1.72	1.78	1.80
	3.0	1.37	1.80	1.56	1.54	1.58	1.57	1.60
100	0.0	252.53	285.76	343.36	288.67	349.56	342.89	340.05
	0.2	214.23	197.04	204.31	189.63	201.71	201.65	204.39
	0.4	106.90	77.72	111.47	109.63	112.71	111.04	108.51
	0.6	48.50	65.42	58.22	55.10	58.81	59.07	60.97
	0.8	32.43	35.82	35.65	33.92	36.51	35.03	34.88
	1.0	19.07	17.48	18.11	19.48	20.51	20.96	21.08
	1.2	10.43	13.82	11.45	13.19	13.94	13.64	13.37
	1.4	7.57	7.98	9.99	8.25	9.28	9.14	9.05
	1.6	6.43	6.22	6.51	5.97	6.45	6.52	6.41
	1.8	5.87	4.78	4.65	4.49	4.68	4.74	4.77
	2.0	3.80	3.98	3.63	3.63	3.65	3.70	3.66
	2.2	2.20	2.66	3.34	2.62	2.80	2.93	2.91
	2.4	2.73	2.34	2.28	2.51	2.39	2.35	2.37
	2.6	1.77	1.92	2.03	2.03	2.11	2.03	2.02
	2.8	1.97	1.64	1.61	1.62	1.70	1.76	1.75
	3.0	1.53	1.32	1.51	1.51	1.54	1.52	1.55
250	0.0	368.30	342.90	351.17	323.11	347.79	351.14	349.57
	0.2	302.23	201.72	225.86	235.58	230.69	228.18	232.14
	0.4	103.83	118.00	121.18	123.20	129.02	128.83	127.03
	0.6	70.67	68.60	57.41	66.44	69.52	67.85	70.62
	0.8	41.27	42.74	36.09	35.30	38.54	39.72	39.98
	1.0	30.90	21.16	22.04	23.92	23.60	23.80	23.63
	1.2	11.13	16.46	12.82	13.96	15.00	15.00	15.32
	1.4	10.80	10.58	9.31	9.37	9.98	9.92	9.99
	1.6	6.47	5.80	6.44	6.06	7.01	6.96	6.87
	1.8	4.73	4.76	4.88	5.46	4.99	5.05	5.00
	2.0	3.33	3.38	3.60	3.58	3.72	3.74	3.79
	2.2	2.90	2.52	2.79	2.99	2.86	2.93	2.97
	2.4	2.37	2.30	2.27	2.41	2.52	2.42	2.41
	2.6	1.80	1.86	1.86	2.04	1.97	2.03	2.01
	2.8	1.60	1.50	1.76	1.72	1.71	1.72	1.72
	3.0	1.37	1.42	1.64	1.51	1.58	1.51	1.52
1000	0.0	328.17	321.28	371.30	370.37	373.70	369.65	366.93
	0.2	350.60	216.02	269.37	253.75	256.90	267.14	262.44
	0.4	122.07	121.30	127.53	131.48	147.11	148.59	150.04
	0.6	107.77	81.86	66.60	81.69	76.43	80.23	82.08
	0.8	33.77	39.68	39.94	47.05	45.32	46.74	46.88
	1.0	26.07	30.54	24.69	24.20	29.12	27.34	27.32
	1.2	12.90	14.84	15.67	15.54	16.31	16.39	16.99
	1.4	9.47	9.36	10.55	11.63	10.47	11.07	10.99
	1.6	7.50	7.62	7.38	7.55	7.35	7.55	7.61
	1.8	4.70	4.86	4.99	5.16	5.30	5.35	5.27
	2.0	2.87	3.96	3.52	3.92	4.05	4.02	4.00
	2.2	2.93	2.30	2.92	3.06	3.07	3.07	3.04
	2.4	1.93	2.26	2.14	2.39	2.45	2.44	2.46
	2.6	1.93	2.20	1.85	1.99	1.96	2.01	2.02
	2.8	1.67	1.78	1.61	1.67	1.66	1.72	1.72
	3.0	1.33	1.56	1.50	1.42	1.51	1.50	1.52

Assuming *δ*=0 with *n* = 30, the ARL values are 214.13, 267.22, 241.56, and 320.85 for sample sizes 30, 50, 100, and 200, respectively. As *δ* increases to 1.00, there is a significant decrease in the ARL values, i.e., 16.73, 17.58, 14.53, and 16.18. For *n* = 50 and *δ* = 0, the ARL values with *m* 30, 50, 100, and 200 are 252.33, 367.98, 314.41, and 312.59, respectively. As the *δ* increases to 1.00, there is a noticeable decrease in the ARL values to 17.17, 20.38, 20.39, and 19.89. For n=100 and *δ*=0, the ARL values for m=30, 50, 100, and 200 are 252.53, 285.76, 343.36, and 288.67, respectively. As *δ* increases to 1.00, the ARL values decrease to 19.07, 17.48, 18.11, and 19.48. With *δ* = 2.00, the ARL values further decreased (3.80, 3.98, 3.63, 3.63) and assuming n=250 with *δ* = 0, the ARL values for *m*=30, 50, 100, and 200 are 368.30, 342.90, 351.17, and 323.11, respectively. For *n* = 1000 and *δ* = 0, the ARL values for sample sizes 30, 50, 100, and 200 are 328.17, 321.28, 371.30, and 370.37, respectively. As *δ* increases to 1.00, there is a decrease in the ARL values to 26.07, 30.54, 24.69, and 24.20, indicating the sensitivity of the control chart to detect shifts. With *δ* = 2.00, the ARL values continue to decrease (2.87, 3.96, 3.52, 3.92), emphasizing the sensitivity of the control chart. A *δ* of 3.00 leads to further reductions in the ARL values (1.33, 1.56, 1.50, 1.42), showcasing the enhanced ability to detect shifts. Thus, as the number of observations approaches 1000, the target in-control ARL of 370 is effectively achieved.

### 3.4 Results for the generalized exponential distribution

Table 4, Table 5, Table 6 tabulate the ARL values for the generalized exponential distribution with shape parameter values of 0.8, 1, 1.5, and the rate parameter of 1. Different combinations of simulations (*m*) and observations (*n*) are taken and the ARL values are calculated for different shift scenarios. The goal is to achieve the IC ARL of 370 assuming k=3. The IC ARL at shift zero acts as the baseline. Furthermore, OOC ARLs are also calculated to assess the ability of the chart for OOC situations.

**Table 4:** ARL values for different combinations of *n*, *m*, and *δ* for Generalized exponential distribution with *α* = 0.8 and *λ* = 1.

		Gen exponential distribution (α=0.8,λ=1,k=3)						
	*m*	30	50	100	200	1000	5000	10000
*n*	*δ*	ARL	ARL	ARL	ARL	ARL	ARL	ARL
5	0.0	127.30	91.50	101.39	99.13	102.44	113.80	107.00
	0.2	235.30	187.18	206.15	196.56	182.33	189.60	180.00
	0.4	369.50	299.20	342.93	326.58	384.49	398.00	375.80
	0.6	669.47	588.48	579.00	684.76	561.25	587.60	562.80
	0.8	1566.27	1183.74	1370.68	1499.90	1462.60	1437.40	1409.60
	1.0	2595.90	2871.10	3004.12	3229.75	3263.41	3223.40	3793.20
	1.2	6346.30	6982.08	6416.53	6394.15	6115.85	6805.80	6406.60
	1.4	12668.10	14868.60	10215.07	14055.10	14042.48	14908.00	17141.00
	1.6	17766.27	29411.68	23696.53	26612.09	32839.28	24482.60	29342.20
	1.8	54792.27	62777.94	51652.22	43319.53	60225.39	58598.00	50346.20
	2.0	146420.20	94236.56	92452.39	111469.18	116354.40	134365.00	116242.40
	2.2	369197.50	191918.82	218638.53	249208.64	670151.06	400106.40	482933.80
	2.4	454837.03	486256.24	467445.20	481810.33	473494.20	259974.80	763030.20
	2.6	783706.60	1126099.24	1063845.69	1216383.12	1055399.00	1395642.60	1611826.00
	2.8	2594347.83	2349584.72	2139946.50	2766024.91	6945702.60	5173965.00	1743176.80
	3.0	5052089.70	6013067.86	6210624.70	5128014.37	6611963.00	6503289.00	6700874.00
30	0.0	222.27	296.92	243.10	240.27	228.60	222.32	220.05
	0.2	370.20	351.36	297.58	354.58	417.11	403.95	397.32
	0.4	611.17	738.02	527.82	709.02	744.46	693.27	719.68
	0.6	1357.40	1116.38	1375.19	1206.75	1112.26	1142.97	1152.33
	0.8	1391.23	1613.40	1782.50	1597.19	1522.64	1496.93	1509.91
	1.0	1094.43	1467.04	1408.42	1718.51	1647.64	1507.20	1543.43
	1.2	1133.90	918.50	1161.02	1275.43	1277.73	1211.01	1248.49
	1.4	1012.00	987.04	863.09	907.42	869.89	900.17	897.15
	1.6	619.00	587.00	681.60	642.69	666.62	649.30	652.93
	1.8	406.30	387.02	495.84	451.00	469.89	475.11	459.80
	2.0	321.53	286.28	288.62	317.57	328.92	347.16	336.92
	2.2	220.73	231.02	238.39	261.86	240.99	251.01	248.20
	2.4	157.27	178.12	210.29	183.33	183.37	184.36	182.17
	2.6	127.30	151.60	134.96	145.74	138.82	137.01	139.91
	2.8	110.90	101.18	111.41	102.42	104.41	106.20	106.71
	3.0	88.73	63.38	70.72	82.21	80.41	80.82	82.75
30	0.0	301.43	244.26	239.77	249.45	280.51	264.56	265.04
	0.2	537.43	448.04	401.83	448.44	432.99	435.39	443.40
	0.4	523.50	649.26	624.76	691.79	649.95	655.88	663.83
	0.6	762.20	872.64	770.94	815.31	743.79	800.59	778.37
	0.8	665.37	744.02	651.60	743.31	685.70	707.13	700.28
	1.0	428.63	546.42	499.21	541.85	532.66	552.67	544.72
	1.2	433.63	385.20	380.24	370.93	392.77	386.74	384.68
	1.4	261.57	205.06	298.26	259.35	257.74	263.00	263.87
	1.6	179.93	223.40	181.98	188.21	177.01	181.25	185.05
	1.8	111.03	129.16	121.57	133.72	129.44	129.17	133.76
	2.0	88.80	103.70	73.28	90.06	92.49	97.57	95.63
	2.2	48.33	78.72	77.74	63.46	66.86	68.73	70.54
	2.4	81.80	48.80	51.90	48.80	53.79	51.09	53.32
	2.6	37.40	39.20	41.21	37.34	40.40	39.26	39.38
	2.8	29.77	29.78	31.79	36.42	30.87	30.88	30.82
	3.0	20.70	22.86	21.43	22.86	24.66	24.68	23.89
100	0.0	304.67	209.08	319.92	284.82	308.33	303.21	310.71
	0.2	441.13	492.60	447.66	445.16	458.22	441.37	448.11
	0.4	762.10	475.96	632.49	521.41	555.92	527.30	533.94
	0.6	576.80	537.54	469.26	481.80	465.83	471.56	462.48
	0.8	427.10	296.22	309.07	356.86	350.98	340.78	342.90
	1.0	266.07	227.46	261.55	246.47	224.45	233.26	234.32
	1.2	231.70	178.34	151.77	184.29	156.69	158.50	162.47
	1.4	127.90	73.34	109.55	107.24	107.67	107.96	108.39
	1.6	80.73	66.12	74.83	77.47	77.99	77.62	75.07
	1.8	50.23	44.20	68.57	45.65	53.30	54.19	54.43
	2.0	33.27	30.84	42.32	37.33	38.87	39.03	39.30
	2.2	30.33	28.92	37.97	29.33	30.00	28.60	28.84
	2.4	19.23	20.92	23.15	20.56	21.39	21.85	22.00
	2.6	16.90	15.70	19.21	17.32	16.60	16.43	16.88
	2.8	11.33	10.20	12.17	12.83	13.00	13.17	12.97
	3.0	10.73	11.58	11.02	10.68	10.78	10.27	10.37
250	0.0	246.93	391.92	338.04	343.72	348.62	342.45	345.23
	0.2	371.03	423.24	405.98	345.27	413.86	415.83	413.69
	0.4	464.13	481.88	412.56	407.66	389.18	384.27	386.37
	0.6	232.40	266.72	297.99	278.21	290.99	296.32	294.98
	0.8	220.67	190.40	199.50	198.98	212.63	203.38	196.70
	1.0	124.37	141.16	110.15	138.55	137.13	131.07	132.77
	1.2	97.97	83.38	91.41	73.86	88.53	88.63	87.81
	1.4	55.60	72.24	52.62	62.20	58.57	58.33	60.06
	1.6	43.13	44.92	42.65	36.82	42.51	41.60	41.42
	1.8	19.80	31.80	26.29	28.78	31.52	29.32	30.10
	2.0	23.50	21.94	22.68	22.14	22.05	22.02	21.24
	2.2	17.93	14.36	17.95	14.01	16.09	15.96	15.82
	2.4	13.07	10.66	12.20	12.93	11.99	12.24	11.95
	2.6	10.30	10.82	8.17	9.56	9.51	9.21	9.40
	2.8	7.63	7.82	6.69	6.45	7.33	7.19	7.37
	3.0	5.23	4.46	5.57	6.05	5.51	5.89	5.91
1000	0.0	365.53	375.72	381.88	373.29	365.34	368.00	361.50
	0.2	337.23	393.14	384.32	402.64	375.56	369.73	379.75
	0.4	269.93	279.26	289.09	287.84	304.60	296.98	295.43
	0.6	205.87	147.46	164.85	196.88	208.16	206.19	201.87
	0.8	144.37	110.06	112.47	126.02	140.81	132.53	134.65
	1.0	58.40	99.22	97.39	84.51	82.34	85.75	86.86
	1.2	71.17	56.84	48.23	54.26	58.25	58.84	57.64
	1.4	40.17	44.64	49.34	37.00	38.87	39.85	39.37
	1.6	26.77	30.78	29.71	26.21	28.29	27.18	27.26
	1.8	18.73	24.42	21.98	19.56	19.56	19.18	19.63
	2.0	12.70	12.00	14.90	14.84	14.65	14.42	14.06
	2.2	9.17	10.02	9.47	10.80	10.66	10.60	10.61
	2.4	7.63	8.78	7.25	8.37	7.98	8.15	8.09
	2.6	6.40	6.02	5.53	6.28	6.00	6.25	6.34
	2.8	8.17	4.56	4.80	4.76	4.85	4.92	4.98
	3.0	3.87	4.68	3.76	4.08	4.14	3.99	4.05

**Table 5:** ARL values for different combinations of *n*, *m*, and *δ* for Generalized exponential distribution with *α* = 1 and *λ* = 1.

		Gen exponential distribution (α=1,λ=1,k=3)						
	*m*	30	50	100	200	1000	5000	10000
*n*	*δ*	ARL	ARL	ARL	ARL	ARL	ARL	ARL
5	0.0	106.57	114.36	99.50	101.45	104.40	183.40	105.04
	0.2	252.77	204.48	234.11	239.34	262.20	211.20	190.68
	0.4	416.23	494.10	420.98	434.98	325.20	314.00	402.22
	0.6	1411.97	1148.10	962.57	977.38	448.60	747.40	725.42
	0.8	1406.13	2345.84	2440.41	2334.66	1339.80	1883.00	1338.10
	1.0	3868.73	5116.26	4701.47	4675.22	8132.20	5173.60	2767.29
	1.2	10302.07	9411.78	11940.35	12023.78	10086.20	12801.20	6122.35
	1.4	34693.77	36037.08	27685.56	29786.16	8904.80	17122.00	11903.13
	1.6	66113.30	50624.82	63620.34	65784.76	56874.80	98676.40	27405.14
	1.8	196352.80	138891.56	143794.33	143794.33	44547.40	103272.60	49914.78
	2.0	222300.90	351273.92	311976.14	311976.14	392269.40	331488.80	116016.70
	2.2	744464.20	957516.70	735435.20	735435.20	1460825.20	600358.00	236364.74
	2.4	1908072.00	2031631.64	2044075.20	2044075.20	2102233.00	3262944.00	520972.51
	2.6	4888776.70	4773259.74	4553745.85	4553745.85	5923970.80	8493413.80	1089265.64
	2.8	11249500.00	9037212.52	12034520.02	12034520.02	17932466.00	7077457.00	2355726.34
	3.0	26110320.00	26646192.98	33560596.57	33560596.57	22635280.00	42264171.20	52451679.28
30	0.0	183.97	245.96	236.74	218.13	241.43	239.29	234.33
	0.2	479.13	438.64	444.36	508.05	460.27	454.35	451.87
	0.4	877.00	885.66	740.36	803.16	848.61	802.67	790.43
	0.6	1195.17	1013.94	1207.38	1242.31	1136.53	1106.87	1095.77
	0.8	714.07	1142.94	1083.88	1047.18	1078.83	1027.00	1063.55
	1.0	897.43	1037.72	713.67	753.82	777.04	767.94	784.93
	1.2	618.67	416.18	531.92	485.01	499.84	539.00	536.82
	1.4	417.33	404.16	308.47	359.02	353.82	349.47	341.52
	1.6	191.37	225.44	215.65	255.28	240.90	234.50	236.16
	1.8	227.87	200.42	171.98	144.57	169.52	166.59	168.31
	2.0	105.57	113.12	106.69	124.18	113.78	114.22	116.14
	2.2	77.87	76.12	89.58	78.58	80.93	81.17	83.22
	2.4	54.60	64.28	62.51	62.31	58.47	61.34	60.96
	2.6	52.43	45.08	39.93	50.30	45.02	45.44	46.00
	2.8	34.00	30.02	39.39	30.13	35.61	34.53	35.18
	3.0	22.57	22.46	26.06	24.61	27.79	27.19	26.41
50	0.0	197.50	302.74	244.78	277.77	270.86	273.46	276.59
	0.2	504.07	424.28	523.02	421.95	481.12	472.88	478.28
	0.4	869.83	647.48	719.11	606.50	672.19	662.96	654.28
	0.6	533.07	658.38	611.92	659.90	636.63	622.55	628.32
	0.8	627.73	473.16	498.07	431.91	478.74	465.30	467.53
	1.0	344.30	295.20	312.09	286.49	295.33	304.52	306.60
	1.2	139.17	239.50	183.73	194.34	202.88	198.44	199.66
	1.4	147.23	166.02	124.33	149.71	127.50	133.03	132.55
	1.6	76.40	73.58	73.81	84.33	90.61	89.47	87.82
	1.8	69.67	59.34	55.18	65.37	64.66	60.37	60.41
	2.0	45.33	53.34	47.44	36.09	40.76	42.75	42.10
	2.2	28.47	24.84	35.30	27.23	30.96	31.35	30.93
	2.4	19.17	20.10	20.06	21.91	23.02	23.06	23.21
	2.6	18.10	20.22	17.57	17.83	17.48	17.68	17.30
	2.8	15.77	13.98	13.61	14.00	13.64	13.40	13.18
	3.0	8.20	8.76	9.81	10.03	10.37	10.31	10.33
100	0.0	283.93	330.18	317.70	326.19	317.06	310.55	319.33
	0.2	583.00	399.40	478.44	465.63	439.85	443.89	466.29
	0.4	492.33	392.20	400.48	447.01	468.17	468.68	477.78
	0.6	335.03	406.84	362.45	326.62	356.98	354.65	358.14
	0.8	262.37	194.40	224.44	226.85	226.07	240.21	233.22
	1.0	143.43	158.20	159.25	157.64	140.94	148.40	145.58
	1.2	45.47	91.34	98.48	95.63	95.15	94.63	91.91
	1.4	67.77	51.32	53.57	58.66	60.97	60.34	62.16
	1.6	41.37	39.22	45.03	43.24	41.81	42.47	41.61
	1.8	21.33	24.54	25.41	30.64	28.57	28.54	29.03
	2.0	24.23	19.16	23.19	23.15	20.62	20.40	20.65
	2.2	17.77	14.04	15.24	13.39	14.66	14.80	15.02
	2.4	14.10	9.16	12.52	12.05	11.41	11.42	11.27
	2.6	7.03	9.82	8.30	8.36	8.61	8.51	8.61
	2.8	6.97	6.88	7.64	7.19	6.58	6.79	6.78
	3.0	6.00	5.70	4.69	5.21	5.27	5.40	5.41
250	0.0	345.13	312.12	332.32	323.28	342.77	351.75	343.14
	0.2	504.77	543.72	355.69	436.68	394.27	419.82	418.17
	0.4	338.27	317.18	367.32	352.75	370.38	337.00	344.44
	0.6	212.57	223.56	198.51	212.60	236.66	226.62	228.12
	0.8	131.27	161.52	133.81	131.03	154.09	138.29	141.61
	1.0	90.80	94.48	89.19	84.76	84.06	88.28	86.38
	1.2	61.10	51.48	59.12	49.43	56.39	55.64	55.76
	1.4	36.73	29.96	38.98	39.43	36.00	36.95	36.24
	1.6	31.53	19.74	23.28	25.31	22.98	25.25	24.61
	1.8	18.50	16.76	15.04	17.90	17.89	17.39	17.42
	2.0	12.27	11.02	12.16	13.16	12.25	12.60	12.39
	2.2	9.60	8.00	8.60	8.40	9.10	9.25	9.13
	2.4	7.03	6.46	7.31	6.99	7.09	6.90	6.90
	2.6	5.87	6.16	5.54	5.86	5.23	5.38	5.41
	2.8	5.40	3.66	4.02	3.88	4.08	4.19	4.21
	3.0	3.13	3.58	3.90	3.15	3.36	3.49	3.47
1000	0.0	357.67	334.24	367.48	372.86	366.32	361.55	367.91
	0.2	350.70	291.84	348.62	336.60	358.08	368.06	358.96
	0.4	289.13	262.30	222.60	251.34	256.10	263.72	260.51
	0.6	120.20	138.26	138.77	173.83	155.61	163.00	159.93
	0.8	87.07	117.12	84.47	99.46	95.89	98.71	97.30
	1.0	67.20	60.48	60.13	60.19	60.66	59.60	60.47
	1.2	44.00	48.80	42.57	36.72	38.38	38.45	38.84
	1.4	28.53	23.66	24.69	26.41	24.38	25.20	25.37
	1.6	23.37	20.30	16.15	17.44	17.89	16.64	17.16
	1.8	7.97	10.42	11.96	10.84	12.22	12.09	11.91
	2.0	6.40	7.02	8.35	8.83	8.49	8.38	8.58
	2.2	6.70	5.40	6.50	6.49	6.38	6.45	6.36
	2.4	5.93	4.18	4.59	5.32	4.76	4.85	4.90
	2.6	4.13	3.14	4.16	3.87	4.01	3.85	3.83
	2.8	2.77	3.36	2.93	3.26	3.02	2.99	3.12
	3.0	2.67	2.66	2.51	2.63	2.53	2.55	2.57

**Table 6:** ARL values for different combinations of *n*, *m*, and *δ* for Generalized exponential distribution with *α* = 1.5 and *λ* = 1.

		Gen exponential distribution (α=1.5,λ=1,k=3)						
	*m*	30	50	100	200	1000	5000	10000
*n*	*δ*	ARL	ARL	ARL	ARL	ARL	ARL	ARL
5	0.0	148.10	131.12	130.74	123.99	120.00	128.00	101.00
	0.2	250.83	310.06	263.90	251.92	286.20	206.20	256.00
	0.4	588.27	526.58	639.88	495.49	315.20	564.70	425.90
	0.6	1384.00	904.90	1026.81	1058.06	763.40	548.20	1018.00
	0.8	2259.63	2620.54	2072.91	2511.09	1254.00	665.50	2839.90
	1.0	4652.80	4760.82	5784.71	5147.64	4484.60	2941.70	3436.70
	1.2	8778.03	12175.88	11418.73	11576.09	17804.00	5149.30	5995.70
	1.4	19993.57	21770.10	19360.55	23350.65	26960.80	29397.50	22831.40
	1.6	28529.90	45140.90	48789.55	45955.65	61604.60	45653.20	37045.40
	1.8	117351.30	86712.74	116827.66	109482.86	156713.40	193534.30	88172.30
	2.0	252313.90	218589.34	266279.89	273353.74	420514.00	398315.00	318291.30
	2.2	736011.70	672610.80	609767.57	585457.73	913802.80	819659.70	1487524.10
	2.4	1590401.00	1408568.52	1392316.38	1374458.66	1711471.60	1083322.00	2270640.10
	2.6	2787383.00	2996861.64	3299257.55	3221497.26	4608160.40	4433888.00	6258963.30
	2.8	8472578.00	8448942.38	7295099.73	7505769.57	17949122.20	9779936.00	10109355.80
	3.0	18524710.00	16159501.42	19454961.11	18954374.11	41111504.60	15912400.00	29405318.00
30	0.0	224.90	212.94	213.83	259.15	256.93	253.21	256.58
	0.2	565.03	588.18	474.07	561.46	539.40	540.77	526.42
	0.4	747.97	860.48	751.64	727.44	755.90	754.96	780.11
	0.6	845.80	656.18	684.26	596.92	606.60	639.80	642.42
	0.8	287.40	376.92	397.47	364.01	387.68	394.44	385.45
	1.0	168.63	217.86	246.40	228.12	227.08	224.75	221.62
	1.2	121.17	152.92	136.32	136.75	127.45	133.16	129.74
	1.4	65.17	79.92	76.56	84.92	84.62	82.52	82.26
	1.6	51.17	57.94	55.54	55.53	50.98	51.83	51.16
	1.8	34.13	29.58	31.09	32.43	34.77	34.44	34.44
	2.0	25.33	25.10	23.55	24.77	26.08	23.66	23.49
	2.2	17.30	16.52	14.71	18.51	17.17	16.80	16.67
	2.4	12.93	9.30	11.54	11.28	11.69	12.31	12.21
	2.6	11.13	8.10	9.04	9.30	9.19	9.14	9.22
	2.8	7.43	6.54	6.90	7.74	7.14	7.11	7.14
	3.0	4.60	5.90	5.73	5.26	5.65	5.60	5.53
50	0.0	209.07	315.42	275.27	321.10	299.43	284.33	288.25
	0.2	475.83	445.44	491.10	580.73	509.26	510.86	506.63
	0.4	700.20	625.70	601.99	530.96	521.75	523.74	523.81
	0.6	404.53	372.78	326.03	293.05	336.00	344.15	341.15
	0.8	141.60	236.76	216.38	165.97	190.00	188.39	192.23
	1.0	109.77	102.40	103.67	116.65	115.81	109.73	111.74
	1.2	66.77	62.78	65.51	56.73	60.47	65.84	65.55
	1.4	42.10	41.42	37.21	44.03	40.38	39.17	41.13
	1.6	24.87	27.90	22.56	25.14	26.10	26.58	26.61
	1.8	14.43	14.24	20.36	18.05	17.71	17.40	17.49
	2.0	8.07	14.90	15.71	12.39	12.53	12.41	12.05
	2.2	10.73	8.38	9.83	8.67	9.02	8.94	8.75
	2.4	6.13	6.98	7.43	6.36	6.45	6.49	6.52
	2.6	4.23	4.60	5.25	5.40	5.00	4.98	5.04
	2.8	2.90	3.68	4.96	4.09	3.95	4.00	3.92
	3.0	3.53	3.32	3.59	3.06	3.29	3.25	3.28
100	0.0	325.10	300.06	339.86	328.37	331.49	319.90	325.79
	0.2	397.13	502.16	458.02	444.12	441.62	455.88	455.43
	0.4	299.90	269.12	325.84	407.95	360.19	356.57	355.52
	0.6	225.30	201.28	206.59	206.28	214.56	198.09	204.80
	0.8	111.60	113.02	105.07	107.33	110.86	108.34	111.94
	1.0	70.90	61.24	74.88	62.45	62.46	63.66	63.54
	1.2	42.87	32.62	40.24	35.85	36.21	37.53	38.16
	1.4	28.03	22.42	24.78	23.81	23.76	24.01	23.66
	1.6	17.07	15.62	17.61	15.78	15.12	15.07	15.09
	1.8	11.43	10.34	11.13	9.21	10.32	10.52	10.31
	2.0	9.90	6.80	8.68	7.16	7.29	7.48	7.16
	2.2	5.70	6.26	5.61	5.03	5.66	5.36	5.34
	2.4	4.07	4.72	3.57	3.45	3.99	4.07	4.08
	2.6	3.13	3.16	3.15	3.14	3.30	3.24	3.19
	2.8	2.87	2.96	2.66	2.30	2.55	2.58	2.58
	3.0	2.10	2.22	2.04	2.10	2.14	2.16	2.17
250	0.0	346.87	336.50	301.13	343.78	342.05	346.23	350.79
	0.2	392.27	392.56	401.14	362.58	403.92	390.93	383.80
	0.4	206.37	220.18	224.09	255.90	234.20	249.12	247.38
	0.6	163.37	150.62	121.99	125.80	135.81	137.02	137.71
	0.8	69.80	86.62	71.73	78.14	74.42	73.77	73.80
	1.0	32.30	47.86	45.81	44.08	39.80	42.28	41.72
	1.2	27.77	20.30	25.22	26.23	25.79	25.05	24.66
	1.4	19.37	17.44	14.82	15.58	15.20	15.92	15.30
	1.6	10.47	10.82	9.53	11.32	10.09	10.39	10.37
	1.8	7.27	7.10	7.65	7.32	6.72	6.86	6.99
	2.0	4.83	4.16	6.07	5.02	5.19	5.05	5.03
	2.2	3.10	3.56	3.59	4.08	3.78	3.75	3.71
	2.4	2.63	2.62	2.75	3.11	2.88	2.91	2.89
	2.6	2.57	2.16	2.58	2.29	2.29	2.33	2.35
	2.8	1.70	1.80	2.00	1.80	1.95	1.94	1.93
	3.0	1.37	1.68	1.59	1.67	1.66	1.68	1.68
1000	0.0	334.37	347.36	361.24	376.98	367.54	365.05	362.75
	0.2	333.33	249.40	301.07	366.13	307.70	328.20	325.07
	0.4	154.33	168.64	206.33	197.36	195.54	198.11	192.43
	0.6	81.10	89.62	106.84	112.84	105.54	100.84	102.27
	0.8	67.07	49.34	57.68	57.20	54.10	54.52	54.30
	1.0	20.93	32.90	29.13	29.98	30.82	31.41	31.00
	1.2	15.00	15.64	16.87	17.01	18.18	18.49	18.28
	1.4	10.00	10.18	12.51	9.54	11.50	11.44	11.40
	1.6	6.40	7.56	8.07	7.91	7.31	7.48	7.59
	1.8	4.80	4.98	5.40	5.15	5.44	5.28	5.25
	2.0	5.03	3.66	3.69	3.51	3.66	3.80	3.74
	2.2	3.63	3.24	2.90	2.77	2.94	2.90	2.87
	2.4	1.90	2.04	2.50	2.14	2.37	2.25	2.30
	2.6	1.80	2.04	2.17	1.85	1.84	1.89	1.88
	2.8	1.53	1.60	1.55	1.54	1.65	1.62	1.60
	3.0	1.80	1.50	1.33	1.45	1.45	1.42	1.41

The results reveal that the number of simulations (*m*) influences the ARL values. A smaller number of simulations, such as m=30, exhibit greater unpredictability, particularly with larger shifts, reflecting a potential reduction in sensitivity to process changes. As the number of simulations to m=50, the ARL results approaches to the desired ARL indicating better detection. When the number of simulations is increased to m=100, the ARL values further increased and a larger number of simulations of m=1000, we obtained more consistent values. It is also evident that the IC ARL is influenced by the number of observations (*n*). For smaller values of *n*, the IC ARL tends to be lower, reflecting the chart's sensitivity to detecting shifts. However, as *n* increases, the IC ARL tends to approach the desired ARL. For example, if *n* is set to 5, the IC ARL is relatively low, but as *n* increases to 50, the ARL values also rise and the trend continue as *n* further increases to 100 and 250, with the IC ARL values showing a gradual increase. However, using a large number of observations, n=1000, we successfully achieved our goal of ARL=370. The shifts in the table represent the magnitude of the deviation from the baseline rate parameter. It is noticed that minor shifts will be detected slowly as compared to the large shifts. To reach the desired IC ARL of 370, a compromise between simulations and observations must be reached. The stabilizing trend in ARL values indicate that increasing the number of observations to 1000 is sufficient.

### 3.5 Comparison of results for Weibull and generalized exponential distributions

The ARL values for the generalized exponential distribution with *γ* parameters of 0.8, 1, and 1.5 are examined assuming λ=1.0. Various numbers of simulations (*m*) and number of sample sizes *n* are examined to achieve the desired IC ARL of 370 with a fixed threshold value k=3. To this end, when *n* was set to 1000, all parameter combinations with k=3 achieved the desired ARL=370. Furthermore, Weibull distribution with *γ*= 0.8, 1, and 1.5, and λ=1.0 is also considered for the comparison. It is noticed that adjustments to *k* were required for each shape parameter of the Weibull distribution to achieve the desired ARL target of 370. That is, k=3.6 is required for *γ*=0.8 and *λ*=1. Similarly, using *n*=1000, a threshold value of k=3 is found to be effective for *γ* = 1 and *λ* = 1, and k=2.33 is good for γ=1.5 and λ=1.

This comparison illustrates an intriguing aspect regarding the two distributions' performance. When n=1000, the generalized exponential distribution demonstrated robustness in achieving the desired ARL of 370 by using k=3. The Weibull distribution, on the other hand, needs different threshold values (k) to achieve the target ARL for different shape parameters. Although both distributions are capable of obtaining the desired ARL, the generalized exponential distribution performance is satisfactory with large *n* as the chart does not require different values of *k* for different shape parameters. This feature facilitates control chart construction by maintaining the same *k*. The Weibull distribution, on the other hand requires nuanced adjustments to the threshold value dependent on its shape parameter value, which potentially adds complexity to the management. For the generalized exponential distribution, the chart with n=5 shows very large ARLs, unlike the Weibull distribution, where the ARLs are not as large.

**Figure 1:**
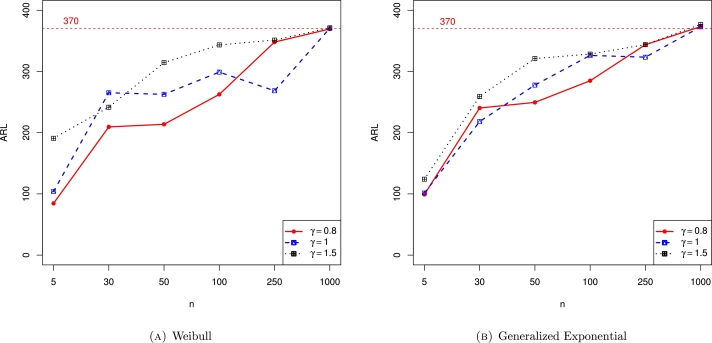
ARL values for Weibull and generalized exponential distributions and there is increasing trend in ARL values. The target ARl is 370 and we obtain the targeted ARL when n approaches to 1000.

**Figure 2:**
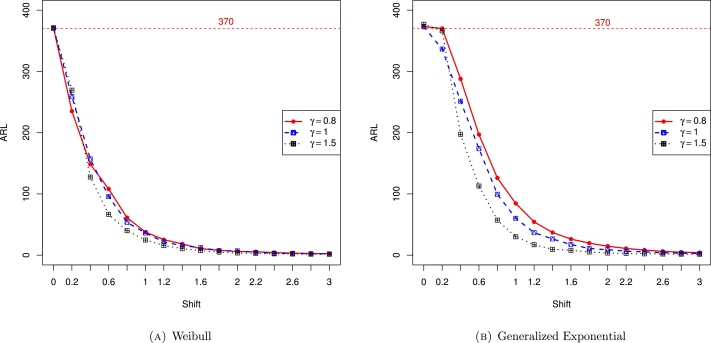
ARL values for Weibull and generalized exponential distributions with varying shifts.

**Figure 3:**
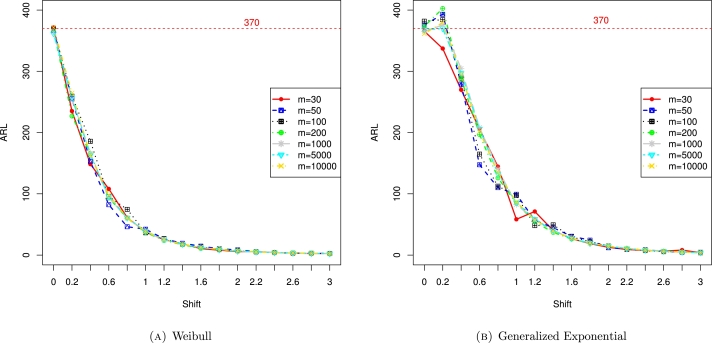
ARL values for Weibull and generalized exponential distributions with varying shifts and number of simulations.

Finally, Fig. 1a depicts the ARL values for the Weibull distribution with different shape parameters. From the figure, it is noticed that as the number of observations (n) increased, the ARL values also approach to the desired ARL value, especially when n=1000. Similarly, Fig. 1b shows the ARL values for the generalized exponential distribution with different shape parameters and the conclusion is the same as in the case of the Weibull distribution. Furthermore, Figs. 2a and 2b depict the ARL values for the Weibull and generalized exponential distributions with varying shifts (*δ*). Assuming n=1000 and $ARL_{0}=370$, the ARL values continuously decrease as *δ* increase. The last two figures, Figs. 3a and 3b, also show the ARL values for the Weibull and generalized exponential distributions with varying shifts (*δ*) and varying simulations *m*. Assuming n=1000 with $ARL_{0}=370$, as *δ* increase, the ARL values continuously decrease.

## 4 Conclusion

This study comprehensively explored the effectiveness of Shewhart control charts for monitoring the mean of Weibull and generalized exponential distributions with a specific focus on the ARL as a pivotal performance metric for evaluating control chart efficiency. Utilizing Monte Carlo simulations, the study validated the precision and reliability to achieve the desired ARL under normal approximation and investigated how non-normal data impacts the performance of Shewhart control charts designed using central limit theorem.

The study particularly investigated the behavior of the $\bar{X}$ control chart when constructed with non-normal datasets by assuming various scenarios related to numbers of observations (*n*) and simulation sizes (*m*). Three non-normal distributions, Weibull, exponential, and generalized exponential, are systematically analyzed. A noteworthy observation from the study emphasized the robustness of the generalized exponential distribution to achieve the target ARL of 370 with k=3 across various parameter combinations when *n* is set to 1000. In contrast, the Weibull distribution requires nuanced adjustments to *k* to meet the target ARL for different shape parameters. Despite both distributions demonstrating the ability to reach the target ARL, the generalized exponential distribution exhibited a more uniform performance. These findings underscore the susceptibility of control chart behavior to factors such as sample size, distribution type, and parameter values. The study also highlights the significance of understanding distribution characteristics and their potential impact on the control chart performance. Ultimately, this research contributes to the advancement of statistical process control methodologies by guiding on control chart threshold for monitoring non-normally distributed processes.

This study can significantly improve managerial decision-making by highlighting the importance of including information on the shape parameter, particularly for the generalized exponential distribution, as this parameter greatly affects the chart's performance. Furthermore, it is advisable to use a large sample size to validate the CLT. The findings indicate that the generalized exponential-based chart is not suitable for small Phase-I datasets, such as when n=5. However, the generalized exponential chart is more robust compared to the Weibull distribution chart because it requires only a single constant for the control limit width. In contrast, the Weibull chart necessitates multiple constants depending on the shape parameter. The information on the shape parameter for both distributions is crucial. Before implementing these charts, it is essential to carefully select or estimate this parameter based on the specific application. The primary limitation of this study is the reliance on the shape parameter knowledge and sample size requirement.

Future efforts should be focused on applying the same technique to other non-normal distributions. Also, this study is concerned with monitoring the mean, and for skewed distributions, the median is more suitable than the mean. Thus, control charts for median monitoring may be studied in the future. Furthermore, exploring various estimation methods for determining the unknown parameters, especially the shape parameter for both distributions, could be beneficial.

## Funding

The authors received no specific funding for the article.

## CRediT authorship contribution statement

**Asad Raza:** Writing – original draft, Software, Methodology, Formal analysis. **Sajid Ali:** Writing – review & editing, Supervision, Methodology, Conceptualization. **Ismail Shah:** Writing – original draft, Validation, Project administration, Investigation. **A.Y. Al-Rezami:** Writing – review & editing, Visualization, Validation, Resources. **Mohammed M.A. Almazah:** Writing – review & editing, Validation, Resources, Funding acquisition.

## Declaration of Competing Interest

The authors declare that they have no known competing financial interests or personal relationships that could have appeared to influence the work reported in this paper.

## Data Availability

Data sharing is not applicable to this article as no new data were created or analyzed in this study. The real dataset is publicly available on the corresponding referenced paper.
